# A register-based observational cohort study on persistent frequent users of emergency services in a Finnish emergency clinic

**DOI:** 10.1186/s12913-019-4723-8

**Published:** 2019-11-21

**Authors:** Jonna M. Levola, Eila S. Sailas, Timo S. Säämänen, Leena M. Turunen, Annika C. Thomson

**Affiliations:** HUS Psychiatry, Helsinki University Hospital and University of Helsinki, Hyvinkää Area, Sibeliuksenkatu 4C, 04400 Järvenpää, Finland

**Keywords:** Emergency service, Register-based cohort study, Psychiatry, Substance use, Substance use disorder, Mental health

## Abstract

**Background:**

The focus of emergency room (ER) treatment is on acute medical crises, but frequent users of ER services often present with various needs. The objectives of this study were to obtain information on persistent frequent ER service users and to determine reasons for their ER service use. We also sought to determine whether psychiatric diagnoses or ongoing use of psychiatric or substance use disorder treatment services were associated with persistent frequent ER visits.

**Methods:**

A cohort (*n* = 138) of persistent frequent ER service users with a total of 2585 ER visits during a two-year-period was identified. A content analysis was performed for 10% of these visits. Register data including International Classification of Primary Care 2 (ICPC-2) –codes and diagnoses were analyzed and multivariable models were created in order to determine whether psychiatric diagnoses and psychosocial reasons for ER service use were associated with the number of ER visits after adjusting for covariates.

**Results:**

Patients who were younger, had a psychiatric diagnosis and engaged in ongoing psychiatric and other health services, had more ER visits than those who were not. Having a psychiatric diagnosis was associated with the frequency of ER visits in the multivariable models after adjusting for age, gender and ongoing use of psychiatric or substance use disorder treatment services. Reasons for ER-service use according to ICPC-2 –codes were inadequately documented.

**Conclusions:**

Patients with psychiatric diagnoses are overrepresented in this cohort of persistent frequent ER service users. More efficient treatments paths are needed for patients to have their medical needs met through regular appointments.

## Background

Emergency room (ER) services are specialized in providing care for patients in need of acute medical attention. Earlier research has found that 8% of ER service users were responsible for 28% of ER visits [1]. This group also uses more hospital services in general [2]. Some patients seek help at the ER repeatedly because it is a place, where they feel safe and perceive their treatment needs are met [3–5]. However, frequent users of ER services are often viewed by staff as difficult [6] or hard to treat [7]. They will often present with psychiatric and substance related issues, social problems such as homelessness, as well as medically unexplained symptoms [8–10]. In many cases, the problems are such that cannot be resolved in ER services, but could benefit from a more comprehensive treatment plan. According to WHO [11], making a treatment plan together with the patient increases the patients’ commitment to treatment, helps to achieve treatment goals and decreases the financial burden of health service use.

While psychiatric symptoms predict higher use of health care services [12], it has also been shown that people with severe mental illness are inadequately treated for their somatic illnesses and are at an increased risk for death from somatic causes [13]. There is a substantial risk, in ER as well as non-emergency services, that somatic complaints are overlooked among these persons. It is unclear whether this is the result of misinterpreting somatic complaints as manifestations of a psychiatric illness or possibly focusing on psychiatric rather than somatic reasons for seeking help. A recent meta-analysis by Sprah et al. [14] also found, that physical comorbid conditions were more common among readmitted psychiatric patients to psychiatric care than among patients with single admissions.

The focus of treatment in the ER is on acute medical crises, but frequent users of ER services often present with various needs. It is unclear, whether these needs are being met through involvement in other services, e.g. psychiatric or substance use services, and whether involvement in these services is associated with less ER service use. More information on this small group of patients, who appear to have unmet needs, is needed when planning comprehensive treatment services and is among the issues that should be addressed as Finland is planning a comprehensive health and social service reform. The objectives of this study were to describe the population who persistently and frequently use ER services, and to determine whether psychiatric diagnoses or psychosocial reasons for ER service as well as ongoing use of psychiatric or substance use services were associated with the number of ER visits among these persistent frequent ER service users.

## Methods

This register-based observational cohort study was carried out at Hyvinkää hospital area in the Hospital District of Helsinki and Uusimaa. The ER services at Hyvinkää hospital provide 24-h medical care to five communities encompassing 196,000 people (May 2017) and both rural and urban areas in Southern Finland. Materials used in this study consist of electronic health records retrieved from the electronic health care record database of the Hospital District of Helsinki and Uusimaa. Analyses were performed anonymously without any information regarding identity. The data was handled, and statistical analyses performed by the third author (TS).

The study was approved by the ethical committee of the Hospital District of Helsinki and Uusimaa (HUS/3914/2017).

### Identification of the cohort

**Fig. 1:**
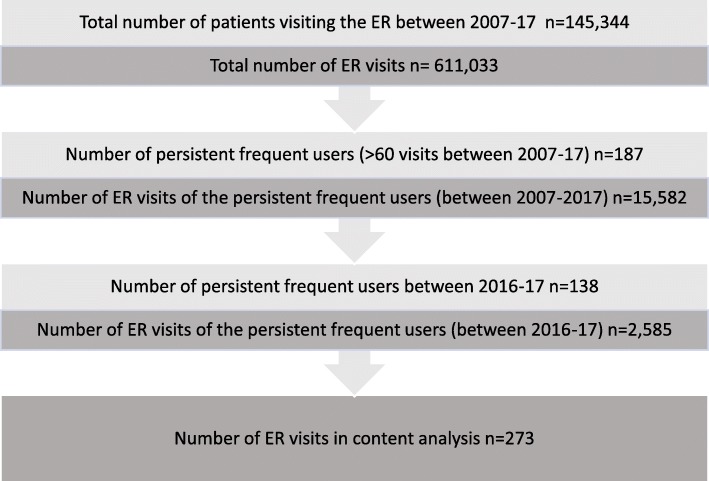
Identification of the study cohort

The process of identifying the study cohort is presented in Fig. 1. ER service use was defined as any contact with the ER department which resulted in admitting the patient into the ER, contacts via phone were not included. Immediately after retrieving the health records, personal identification numbers and names were removed and replaced with study identification numbers.

International Classification of Primary Care 2 (ICPC-2) –codes are used to classify reasons for contacts e.g. in primary care or general practice [16]. Reasons for visiting the ER were analyzed according to ICPC-2 –codes. Psychosocial reasons included psychiatric, substance related and social ICPC-2 –codes. The time of day and week for the ER visits were analyzed in order to determine whether ER visits due to psychosocial reasons occur at distinct times compared to other visits.

After anonymization, the number of ER visits per patient between 2007 and 2017 were extracted to identify the cohort of persistent frequent ER service users. As there is no consensus as to what is defined as “persistent and frequent use of ER services”, patients were identified as persistent frequent users of ER services according to the definition used previously by Saarento et al. [15]. According to this definition, patients were included if they were in the top 10 percentile in the number of ER contacts during 2007–2017. According to this definition, 187 adult individuals with over 60 visits were identified. As ICPC-2 –codes have been used at Hyvinkää hospital systematically only since the beginning of 2016, the study period was determined as 2016–2017. This resulted in 138 persistent frequent ER service users with over 60 visits during 2007–17 and at least one visit during 2016–17. These 138 individuals had a total of 2775 ER visits.

### Content analysis

After identifying the cohort using the ER persistently and frequently (*n* = 138), the health records and the textual content of a random sampling of every tenth ER visit of every individual in the cohort were further analyzed by the fourth author (LT). In the case of six visits no documentation was found (content analysis performed for 267 visits). Socio-demographic variables, substance use and use of other medical and social services during the study period were identified when possible. Information regarding psychiatric and somatic diagnoses were also available for the study period, but through content analysis, only psychiatric diagnoses directly related to the ER visits were determined.

### Statistical analyses

Differences between groups were calculated using the Mann-Whitney U-test (non-normal distribution) for variables having two groups and Kruskal-Wallis for variables with more than two groups. Poisson regression was used in the multivariable models.

For the multivariable analyses, data were grouped in two ways. First, the patients were categorized into those with and without psychiatric diagnosis (psychiatric diagnosis yes/no) (F00– F99; incl. Substance use disorders (SUDs)) according to the International Classification of Diseases, tenth revision (ICD-10) [17]. Second, the data was categorized according to having psychosocial vs. other reasons as the primary reason for the ER visit according ICPC-2 –codes (psychosocial reason for ER visit yes/no). The confounding variables considered were age, gender and ongoing use of psychiatric or SUD treatment services.

First crude models were formed in order to compare the number of ER visits during 2016–2017 between those with vs. without psychiatric diagnoses as well as those having psychosocial vs. other reasons for ER service use (model 1). Then the potential confounders were entered in blocks with age and gender included as covariates in model 2. Last, in model 3, ongoing psychiatric or SUD treatment use was added into the models in order to evaluate the effect of ongoing treatment services on number of ER visits.

Powers and effect sizes were calculated. Post hoc power ranged from 0.06–0.34. Effect sizes are reported in Table 1. Effect sizes (r) were interpreted according to Cohen’s (1988) criteria where > 0.1 reflects small, > 0.3medium and > 0.5 large effect sizes. Data were analyzed with SPSS version 22.0*.* All results were considered statistically significant with a *p*-value of < 0.05.

**Table 1:** Characteristics and differences between groups (Mann-Whitney (U)) of persistent frequent ER service users in Hyvinkää hospital during 2016–2017 (*n* = 138)

	*n*	%	Mean total visits	*p*	Effect size^a^
Age, years					
18–25	15	10.9%	32		
> 25	123	89.1%	17	*0.015*	0.2
Gender					
Male	49	35.5%	19		
Female	89	64.5%	19	0.439	0.1
Employment					
Working	35	25.4%	18		
Not working^b^	84	60.9%	19	0.098	
Any psychiatric diagnosis^a^					
Yes	50	41.3%	25		
No	88	58.7%	15	*0.002*	0.3
ER visit for psychosocial reasons^b^					
Yes	40	29.0%	20		
No	98	71.0%	18	0.163	< 0.1
Ongoing use of services					
Psychiatric services					
Yes	48	34.8%	24		
No	90	65.2%	16	*0.035*	0.2
SUD treatment services					
Yes	10	7.2%	27		
No	128	92.8%	18	0.073	< 0.1
Other health services					
Yes	47	34.1%	23		
No	91	65.9%	17	*0.007*	0.2
Social services					
Yes	18	13.0%	20		
No	120	87.0%	19	0.389	< 0.1

^a^Effect sizes (r) were interpreted according to Cohen > 0.1 small, > 0.3medium and > 0.5 large effect size ^b^On sick-leave or disability pension, or unknown

## Results

**Fig. 2:**
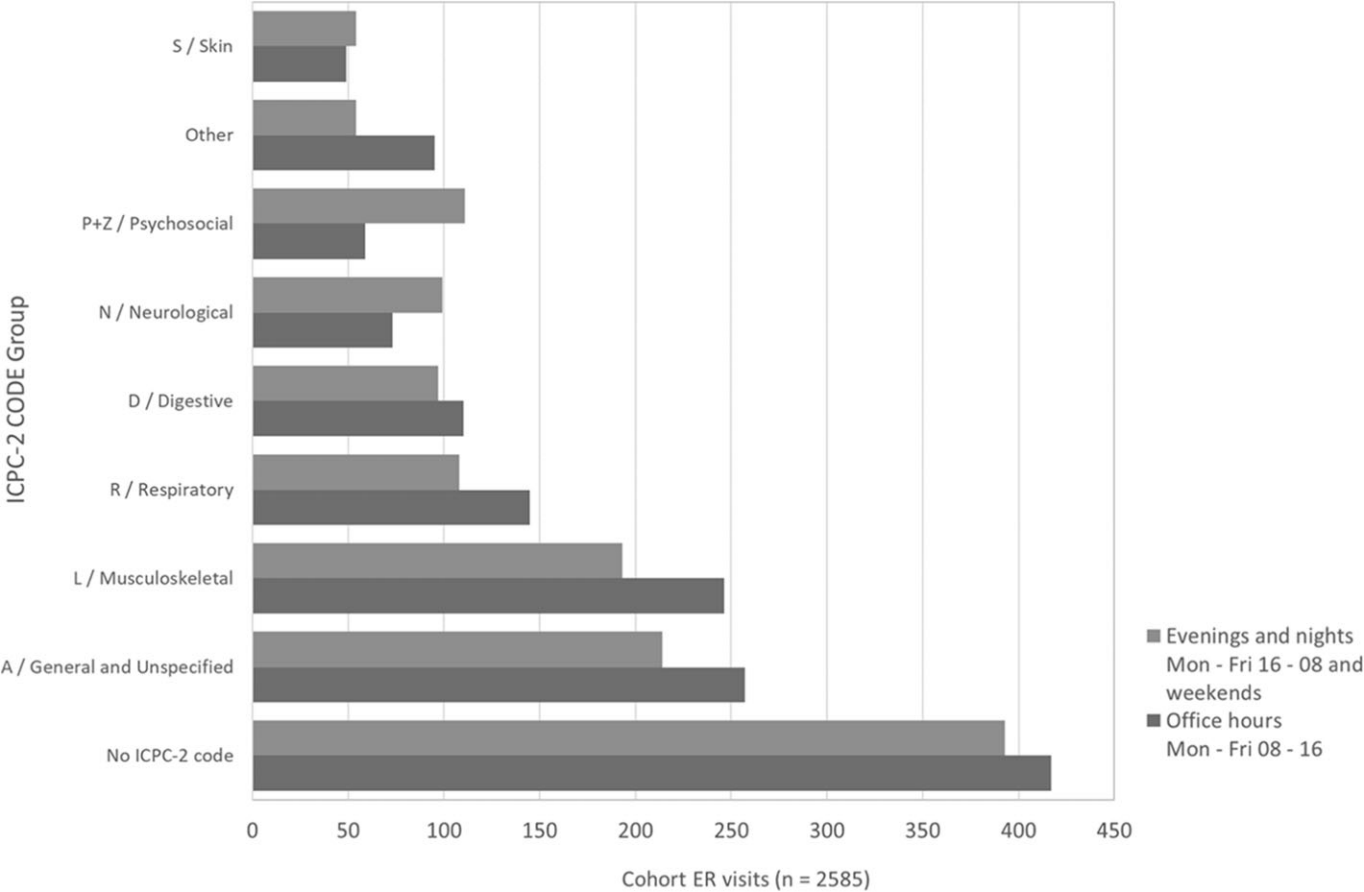
Reasons for ER visits between 2016 and 17 according to time of day and International Classification of Primary Care 2 (ICPC-2) –codes

The characteristics of 138 patients who were identified as persistent frequent users of ER services are displayed in Table 1. The mean age of the patients was 49 (SD 19.84; range 17–94) and the mean number of visits per patient was 18 (SD 14.97; range 1–105 visits). The primary reasons for ER visits according to ICPC-2 -codes are displayed in Fig. 2. The most common reasons for ER service use – when documented – fell under the category “General and unspecified”. The primary reason for the visits could not be interpreted in almost half of all 2775 visits (“No ICPC-2 code” and “General and Unspecified”). During the study period, 29.0% of the cohort (*n* = 40) visited the ER due to psychosocial reasons. Having a psychosocial reason for using ER services was more common during evening, nights and weekends, than during office hours. The most common single psychosocial reason was “Chronic alcohol abuse” (P15; 36.6% of all psychosocial reasons), followed by “Psychological symptom/complaint, other” (P29; 20.5%), “Feeling anxious/nervous/tense” (P01; 14.3%), “Medication abuse” (P18; 11.2%) and “Suicide/suicide attempt” (P77; 9.9%).

### Content analysis

Of the ER visits which underwent content analysis (*n* = 267), social problems, such as economic distress, were reported as the primary cause in 12.7% (*n* = 34) of visits and 5.2% (*n* = 14) visits involved self-harming behaviour. An involuntary referral to a psychiatric inpatient unit was made on a single instance. Of the 138 patients with frequent ER service use, 41.3% (*n* = 57) had any psychiatric diagnosis, the most common of which were alcohol or substance related diagnoses (F1X.XX; 20.3%, *n* = 28) and anxiety-related diagnoses (F4X.XX; 15.9% *n* = 22). Other psychiatric diagnoses were less common (< 6.0%). Psychiatric outpatient care involvement was documented for 34.8% (*n* = 48) of patients and SUD treatment involvement in 7.2% (*n* = 10) of patients.

Substance use was documented in 29.2% (*n* = 78) of all 267 visits with alcohol (23.2% of visits, *n* = 62), benzodiazepines (7.1%, *n* = 19) and opioids (5.2%, *n* = 14) being the most commonly reported substances. Of all visits where substance use was recorded (*n* = 73), use of more than one substance was reported in 23.3% (*n* = 17) of these cases, 13.7% (*n* = 10) were involved in existing SUD treatment and 17.8% (*n* = 13) psychiatric outpatient care.

### Multivariable analyses

Among frequent ER service users, having a psychiatric diagnosis was associated with the number of ER visits (Table 2; IRR 1.59, 95% confidence interval (C.I.) 1.25–1.89). This association persisted after controlling for age, gender and ongoing use of psychiatric or SUD treatment services (IRR 1.50, 95% C.I. 1.17–1.93).

**Table 2:** Association of psychiatric diagnoses and psychosocial reasons for ER service use to number of ER visits (Poisson regression)

	Total *n* = 138	*Model 1*^*a*^		*Model 2*^*b*^		*Model 3*^*c*^	
		*IRR*	*95% C.I.*	*IRR*	*95% C.I.*	*IRR*	*95% C.I.*
Any psychiatric diagnosis^d^	*n* = 50	1.59^***^	1.25–1.89	1.56^***^	1.22–2.00	1.50^**^	1.17–1.93
ER visit for psychosocial reasons^e^	*n* = 40	1.05	0.81–1.36	1.02	0.77–1.33	0.97	0.74–1.28

^**^ *p* < 0.01 ^***^ *p* < 0.001

^a^Association with number of ER-visits, crude model

^b^After adjustment for age and gender

^c^After adjustment for age, gender and use of mental health or substance use services at any time during 2016–2017

^d^Individuals with any F-diagnosis at any time during 2016–2017

^e^Idividuals with ER visit(s) for psychosocial reasons according to ICPC-2 code at any time during 2016–2017

## Discussion

The burden of poor mental health is increasing and affects a substantial part of the population [18, 19] and causes society and the individuals themselves social and economic strain. In the present study, psychiatric diagnoses were notably more common among this population of persistent frequent ER service users than have been reported previously in the general population [20]. Having a psychiatric diagnosis was associated with a higher number of ER visits. This association was statistically significant after adjusting for age, gender and use of psychiatric or SUD treatment services. Having a psychosocial reason for ER service use was not associated with the number of ER visits.

ICPC-2 –codes are designed to reflect subjective reasons for seeking medical attention. Individuals with mental health or substance use related problems do not necessarily seek help from the ER for psychosocial reasons [7]. If an individual e.g. self-mutilates, the reason for seeking help is determined to be somatic, even though the underlying causes have to do with mental health problems. Thus, the finding in this study that psychosocial reasons for ER service use are not associated with the number of visits do not necessarily reflect that psychosocial problems were not present. One must note, however, that people with severe mental illness are inadequately treated for their somatic illnesses, and their somatic complaints must be addressed in concordance with general treatment guidelines.

Ongoing use of psychiatric or SUD treatment services did not markedly change the association between having a psychiatric diagnosis and the number of ER visits in this population of frequent ER users. This may reflect that the reasons for ER service use were not related to psychiatric illness, which is supported by the fact that psychosocial reasons for ER service use were not statistically associated with number of ER visits. An alternative explanation could be, that despite presenting with somatic reasons according to ICPC-2 –codes, the underlying causes are, in fact, related to psychiatric illnesses and ongoing treatment has failed to respond adequately to the patients’ needs.

In the present study, most ER visits due to psychosocial reasons took place outside office hours. This may reflect these patients’ inability to organize their lives according to societal norms and hours. On the other hand, more flexible office hours could help diminish the need for ER service use. Many of the psychosocial reasons for seeking help at the ER are non-urgent [21], which is reflected in this study e.g. by the lack of emergency referrals to psychiatric inpatient care in the context of these visits. Non-urgent reasons for seeking help may, in general, contribute to unnecessary crowding of the ER and thus result in a delay for providing acute care.

Purdie et al. [22] found that a typical frequent visitor (more than six visits during 6 months or 12 visits during 12 months) of ER services was typically an unemployed, single, 48.5-year-old male, who arrived by ambulance. The most common diagnosis was alcohol use disorder (87.5%) followed by epilepsy (31.0%). The persistent frequent users of ER services in our study were more often women than men and younger age was associated with more ER visits. The reason for these differences in relation to previous findings may have to do with the different definitions and attributes of frequent ER service users as opposed to persistent frequent users. In this study, persistent frequent ER use was defined according to frequent use spanning over 10 years. A possible explanation may also be that the ER services in Hyvinkää Hospital encompass both specialized care as well as self-referral services. Women use more health services than men [23, 24], in general, and this may be reflected in the ER when no referral is needed. Frequent visitors at psychiatric emergency services have been described as younger in previous studies [25, 26].

SUDs have previously been reported to be common among frequent ER service users [10]. Diagnoses of SUDs and use of SUD treatment services were quite rare in the present study (*n* = 21, 15% of frequent ER service users), which is somewhat surprising given that alcohol and substance use was documented quite often and with respect to previous research findings [9, 10]. Alcohol use was unsurprisingly the most common substance recorded in the context of ER visits in this study, where ca. 80% of cases with documented substance use involved alcohol. This was an expected finding due to the leading role of alcohol among substances of use in Finland where, on average, 12.1 l of 100% alcohol is annually consumed per every ≥15-year-old inhabitant [27]. Half of this amount is consumed by 10% of the population [28]. Problems with illicit use of prescription medications (benzodiazepines and opioids) were also fairly common in this study, whereas illegal drug use was more infrequent. Opioids and benzodiazepines are frequently seen in polysubstance abuse and are the most common findings in overdose related deaths [29].

Our study has several strengths. The use of register data is reliable and objective with relatively little problems of attrition. Register data did not include information on the use of other ER services elsewhere in Finland and shortcomings in documentation can lead to missing data as was found here in the case of inadequately documented ICPC-2 –codes. Missing data regarding ICPC-2-codes were observed to be more common in the beginning of the study period (beginning of 2016). It was assumed, that this was likely to do with the fact that the use of ICPC-2-codes was implemented from the beginning of 2016 and thus, the missing data had most to do with implementing new policy. Thus, this missing data would not be likely to bias the results. A significant limitation is the selection process of the study cohort, which took into consideration persistent frequent use of ER services during 2007–2017. Some individuals of the cohort had relatively few visits during the study period 2016–17 and no longer belonged to the group of persistent frequent ER service users. This limitation along with the small number of individuals in the study cohort is more likely to underestimate the observed results, rather than emphasize them. The content analysis of patient records allows for more in-depth information on ER visits compared to using register data only, which can be considered a strength.

The results of this study suggest that more diverse treatment paths where psychiatric, substance use and social services are integrated are needed to meet the needs of these frequent users of ER services. Interventions for alcohol and substance use which are applicable to ER settings are warranted. Future research may wish to further characterize this population of frequent ER service users with regard to morbidity and mortality as well as to evaluate how innovative treatment regimens succeed in meeting the treatment needs of this population.

## Conclusions

This study was designed with the purpose of obtaining more information on persistent frequent users of ER services. We found that patients who were younger, had a psychiatric diagnosis and were engaged in ongoing psychiatric and other health services, had more ER visits than those who were not. Psychosocial ICPC-2 reasons for visiting the ER peaked outside office hours. On the basis of these results, we conclude that treatment paths where psychiatric, substance use and social services are integrated must be developed to meet the needs of frequent users of ER services.

## Data Availability

The datasets generated and/or analyzed during the current study are not publicly available due to them containing information that could compromise research participant privacy, but are available from the corresponding author on reasonable request.

## Abbreviations

C.I.: Confidence Interval
ER: Emergency Room
ICD-10: International Classification of Diseases, tenth revision
ICPC-2: International Classification of Primary Care 2
SUD: Substance Use Disorder
